# MERMAID: an open source automated hit-to-lead method based on deep reinforcement learning

**DOI:** 10.1186/s13321-021-00572-6

**Published:** 2021-11-27

**Authors:** Daiki Erikawa, Nobuaki Yasuo, Masakazu Sekijima

**Affiliations:** 1 Department of Computer Science, Tokyo Institute of Technology, 4259–J3–23, Nagatsuta-cho, Midori-ku, Yokohama, Japan; 2 Academy for Convergence of Materials and Informatics (TAC-MI), Tokyo Institute of Technology, S6–23, 2–12–1, Ookayama, Meguro-ku, Tokyo, Japan

**Keywords:** Molecular generation, Lead Optimization, Hit-to-Lead, Monte Carlo Tree Search, Drug Discovery

## Abstract

The hit-to-lead process makes the physicochemical properties of the hit molecules that show the desired type of activity obtained in the screening assay more drug-like. Deep learning-based molecular generative models are expected to contribute to the hit-to-lead process. The simplified molecular input line entry system (SMILES), which is a string of alphanumeric characters representing the chemical structure of a molecule, is one of the most commonly used representations of molecules, and molecular generative models based on SMILES have achieved significant success. However, in contrast to molecular graphs, during the process of generation, SMILES are not considered as valid SMILES. Further, it is quite difficult to generate molecules starting from a certain molecule, thus making it difficult to apply SMILES to the hit-to-lead process. In this study, we have developed a SMILES-based generative model that can be generated starting from a certain molecule. This method generates partial SMILES and inserts it into the original SMILES using Monte Carlo Tree Search and a Recurrent Neural Network. We validated our method using a molecule dataset obtained from the ZINC database and successfully generated molecules that were both well optimized for the objectives of the quantitative estimate of drug-likeness (QED) and penalized octanol-water partition coefficient (PLogP) optimization. The source code is available at https://github.com/sekijima-lab/mermaid.

## Introduction

Approximately 8000 drugs are currently being developed worldwide [1]. From drug discovery to launch, a new drug takes an average of 10 to 15 years to be developed and costs $2.6 billion [1, 2]. Among the drug candidates that enter Phase I clinical trials, less than 12 % are approved by the Food and Drug Administration (FDA) [1]. After the target protein of a therapeutic drug for a disease has been determined, high-throughput screening (HTS) is used to exhaustively test the binding affinity of thousands to hundreds of thousands of compounds to the target protein in the search for hit compounds. Although the number of possible structures of a compound is $$10^{60}$$ and depends on the quality of the compound library to be tested, the hit rate of HTS is approximately 0.1 % [3], which provides an opportunity to discover unexpected hit compounds but also highlights the problem of high experimental cost. To reduce the number of compounds to be tested, virtual screening, a computer-aided drug design (CADD) method for selecting new drug candidates, was proposed in the late 1990s [4]. In virtual screening, compounds that have a high potential to bind to a target protein are ranked in order from a database of thousands to millions of compounds using an evaluation function that expresses the binding affinity calculated by a computer. The compounds narrowed down by the virtual screening are verified by biochemical experiments [5–7], and those that are actually determined to be active proceed to the hit-to-lead. Hit-to-Lead is a stage in early drug discovery where small molecule compounds hit by high-throughput screening (HTS) are processed through certain optimizations to identify promising lead compounds [8]. In addition to simulation, machine learning (ML) methods such as random forests and deep learning have been used in virtual screening [9–12]; however, molecular design methods using generative models are expected to be used in hit-to-lead [13].

The significant progress made in ML in recent years, especially in terms of deep learning, has led to a breakthrough in image processing and natural language processing [14]. Subsequently, various ML models have been applied in the field of molecular design and have shown impressive results [15]. Gomez-Bombarelli et al. [16] used a variational autoencoder (VAE) for molecular design. Representing the molecule as a continuous variable enables us to perform gradient-based optimization in latent space. Considering that simplified molecular input line entry system (SMILES) is a string, which is one representation of molecules, it is natural to adopt recurrent neural networks (RNNs), which are suitable for time-series data such as strings, for molecular design. Segler et al. [17] used long short-term memory (LSTM), which is an RNN, for molecule generation. Although this method showed high validity, it is not suitable for the purpose of generating molecules with desirable properties. This is because LSTM training is only optimized to satisfy SMILES grammar, and the generation process does not consider the properties of molecules. Therefore, we have to repeat the generation process incessantly until we generate the desired molecules. So far, a variety of SMILES-based methods for optimizing specific chemical properties have been proposed [18, 19]. Xiufeng Yang et al. [20] used Monte Carlo tree search (MCTS) to generate desirable molecules with better efficiency than random sampling in RNN-based molecule generation. The aforementioned methods are SMILES-based molecular generative models, and they cannot take a specific molecule as a starting point during optimization tasks. This is because, unlike the case of molecular graphs, sub-SMILES cannot be considered as valid molecules owing to the nature of SMILES grammar.

Graph representation, which is called a molecular graph, is also a useful representation of molecules. Graph representation is easier to understand visually, and checking the valence allows all generated molecules to be valid. Various ML models such as generative adversarial network (GAN) [21] and VAE [22] have been applied to the generation of molecular graphs [23–25]. These methods often outperformed SMILES-based methods in terms of optimization for certain chemical properties, as well as for metrics, such as validity and novelty, for generated molecules. However, handling molecular graphs on a computer is more difficult than SMILES. Because VAE deals with likelihoods explicitly, graph matching is necessary to calculate the loss function, which has a high computational cost [26]. Although GANs do not deal with likelihoods explicitly, the GNN used in the discriminator and generator require a significant computational cost [27]. For these reasons, the molecular graph-based approach can only deal with small molecules [28]. In addition, for both SMILES-based and molecular graph-based approaches, reinforcement learning is used to optimize specific chemical properties; however, the reward model, which is represented by neural networks and is not always accurate(especially in the case of extrapolation) [29], must be retrained for each evaluation function.

**Fig. 1:**
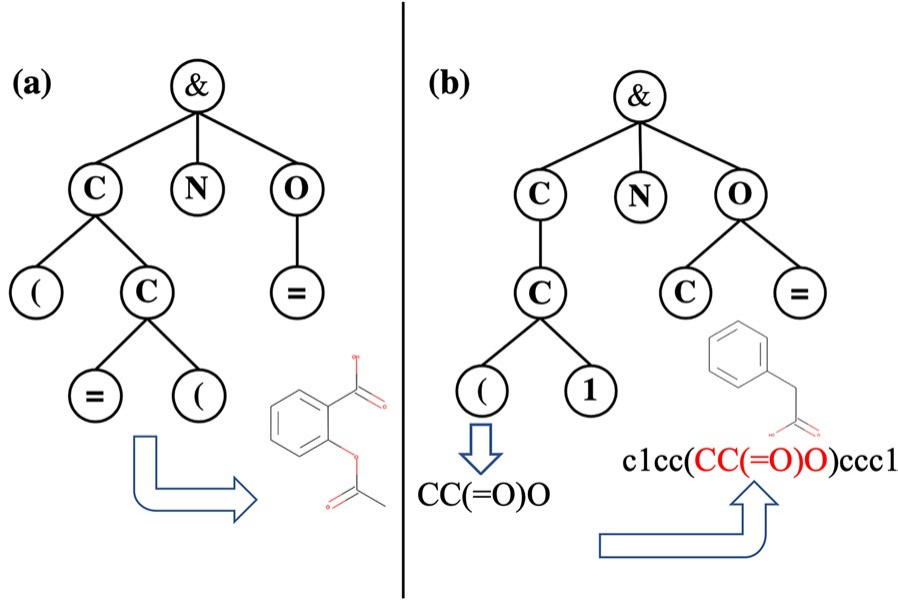
Comparison with existing MCTS-based methods (**a**) Existing MCTS-based molecular generative model such as ChemTS [20]. This model generates full SMILES strings through MCTS. **b** Our model starting from a specific molecule. It generates partial SMILES and replaces a part of the seed SMILES with the generated one

To address these issues, we have introduced *MERMAID*, a generative model based on SMILES using MCTS and RNN. Our model can take a specific molecule as a starting point (Fig. 1), and because we adopt SMILES representation, the restrictions on the size of molecules is not as stringent as the molecular graph-based approach, and our model does not require retraining of the model for each chemical properties.

## Methods

### Optimization of specific molecules

The purpose of this study is to generate the derivatives of specific molecules. The aforementioned MCTS-based generative model cannot start from specific molecules simply, because MCTS only adds nodes to the tail of a search tree. Our proposed method is as follows.

1. Extract partial SMILES strings from the initial point of SMILES.
2. Use MCTS to generate a series of characters representing the partial SMILES strings
3. Replace the extracted partial SMILES strings with the generated partial SMILES

**Fig. 2:**
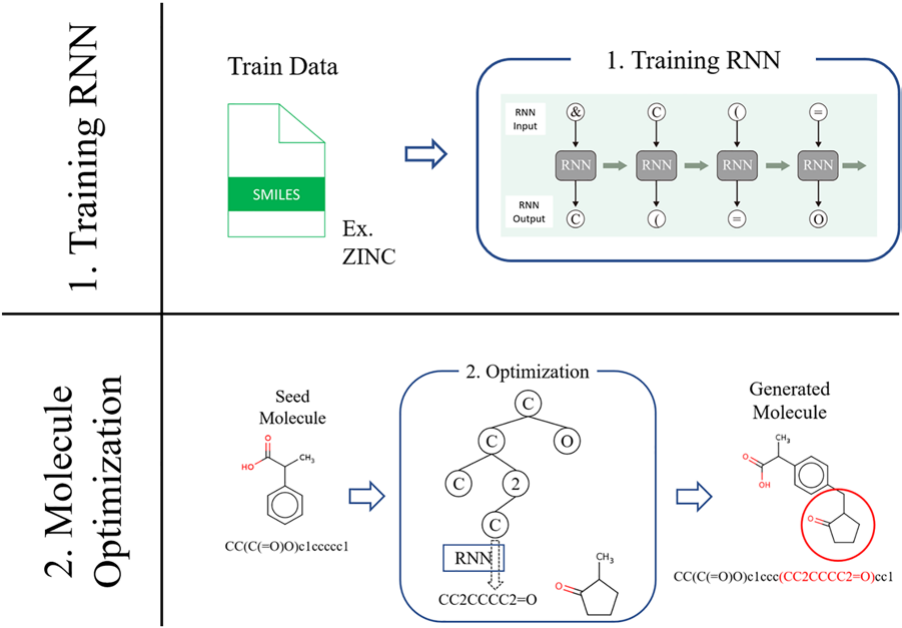
Overview of MERMAID In MERMAID, an RNN model is trained using the SMILES dataset, and MCTS and the RNN model generate SMILES corresponding to the substructure, which are inserted into the SMILES of the seed molecule to generate a new molecule

This method is capable of generating molecules that are obtained by removing or adding a series of zeros or more SMILES characters from SMILES regarded as the starting point (Seed molecule). An overview of our entire methods is given in the Fig. 2. First, the method of (partial) SMILES generation by MCTS will be explained. After that, the RNN and its training procedure will be explained, and details of the substitution procedure used with MCTS will be given.

### MCTS for molecular generation

**Fig. 3:**
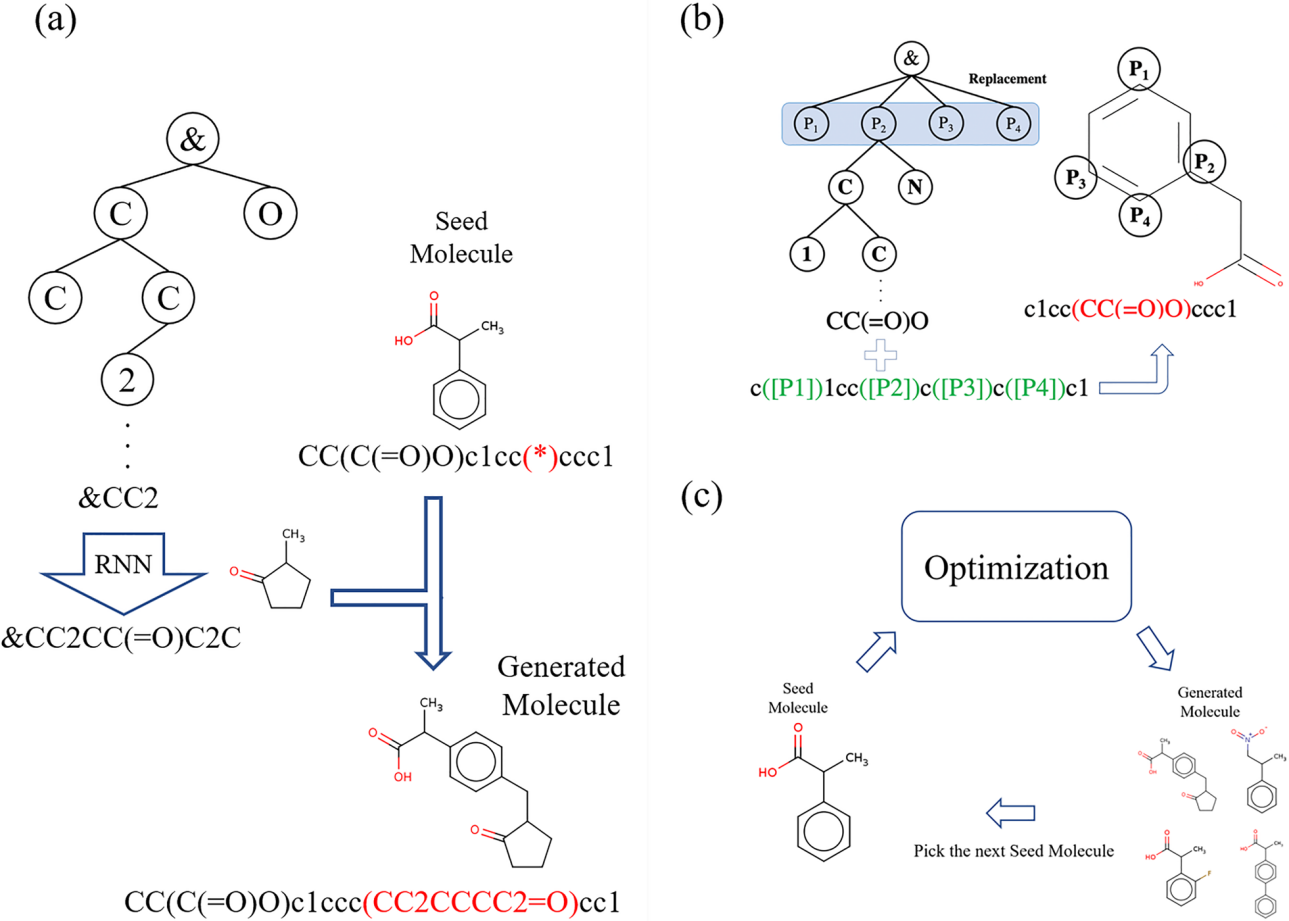
Details of SMILES generation by MCTS (**a**) With the path to the non-terminal node as input, the RNN model generates a complete partial SMILES. Then, by inserting the generated partial SMILES into the seed SMILES, a new SMILES is generated. **b** Selecting seed SMILES that is replaced with the generated one.Nodes under the root node have the information of the part deleted from seed SMILES.The generation of partial SMILES begins from the grandchild nodes of the root node. **c** The optimization cycle is repeated multiple times, replacing one of the generated SMILES with a seed SMILES

MCTS [30, 31] is a model-based reinforcement learning approach used to solve large space planning problems by sampling episodes and constructing search trees. The generation of (partial) SMILES by MCTS is illustrated in Fig. 3(a). A node corresponds to a state $s_i$ and has the value *Q*(*s*), which represents the evaluation of itself and the number of visits *N*(*s*). While sampling episodes, MCTS selects a node from a search tree using tree policies and evaluates a new node by rollout. Rollout is the default policy for simulations without adding new nodes to search tree. The details of Rollout are described later.

In molecular generation, a node corresponds to a character of SMILES; therefore, hence, a path from the root node to leaf node corresponds to a SMILES. The MCTS algorithm includes the following four steps and iterates until some convergence condition is satisfied.

#### Selection

The purpose of this step is to select a node from a search tree for the expansion of nodes. Starting from the root node, the child node of current node is selected based on a tree policy. The tree policy is discussed later. Node selection is repeated recursively until the leaf node is selected.

#### Expansion

The purpose of this step is to expand the current node(the node is selected in Selection step). Some SMILES characters following the current node’s SMILES character are selected from predefined vocabulary.

#### Simulation

The purpose of this step is to evaluate added nodes. The evaluation of non-terminal nodes is a difficult task in reinforcement learning. Therefore, MCTS evaluates non-terminal nodes using the *Rollout* procedure. The *Rollout* procedure expands recursively from evaluating the nodes until the terminal node appears. When the terminal node appears, the path from the root node to the terminal node corresponds to a complete SMILES. Therefore, We can evaluate the path easily using some metrics such as QED, LogP. We used the score as the non-terminal node.

#### Backpropagation

The purpose of this step is to update the value *Q*(*s*) and the number of visit *N*(*s*) of traversed nodes in this episode. Starting from the evaluated node in the Simulation step, the parent node of the current node is updated using the calculated score *r* recursively until the root node appears. The update formulas are defined as follows.

1 $$\begin{aligned} Q(s)\leftarrow & {} \frac{Q(s)N(s) + r}{N(s) + 1} \end{aligned}$$

2 $$\begin{aligned} N(s)\leftarrow & {} N(s) + 1 \end{aligned}$$

Tree policies are important for the performance of MCTS. The upper confidence bound (UCB) score, which is proposed for the multi-armed bandit problem, is often used as the tree policy. Node selection based on the UCB score is as follows.

3 $$\begin{aligned} \pi (s) = \mathop {\hbox {arg max}}\limits _i \left\{ Q(s_i) + 2C_p \sqrt{\frac{\ln {N(s_{p})}}{N(s_i)}}\right\} \end{aligned}$$

where, *Q*(*s*) is the mean estimated value of state *s* for its child nodes, and *N*(*s*) is the number of visits to the state *s*. $s_i$ and $s_p$ are the states of each node *i* and the parent node, respectively. $C_p$ is the hyperparameter of the bias term. The first term corresponds to exploitation, and the second term corresponds to exploration.

The advantage of using UCB score as the tree policy is that the probability of selecting sub-optimal actions converges to zero as the number of iteration tends to infinity under specific conditions (appropriate $C_p$ and the value of reward ranges between 0 and 1). However, it is not possible for the number of iterations to tend toward infinity. Furthermore, the performance of rollout-based algorithms degrades similar to that of other algorithms [32]. Since a character following the SMILES string needs to satisfy SMILES grammar, the number of suitable characters that follow the SMILES string is smaller than the number of vocabularies, which is action space. Therefore, a few characters selected by RNN are considered as actions in our approach. $C_p$ is chosen to be an upper bound of the accumulated reward in practice. However, in this case, $C_p$ is large to an extent that the state space is restricted, i.e., exploration is considered more valuable than exploitation. Therefore, we set $C_p = \frac{1}{\sqrt{2}}$ like other studies [20] that using MCTS.

### Inference of SMILES using RNN

RNN is a type of neural network that propagates information not only in the direction of layers but also that of time series. In this study, LSTM [33], is a type of RNN superior to normal RNN in terms of longer dependency, is used to capture the features of SMILES grammar.

**Fig. 4:**
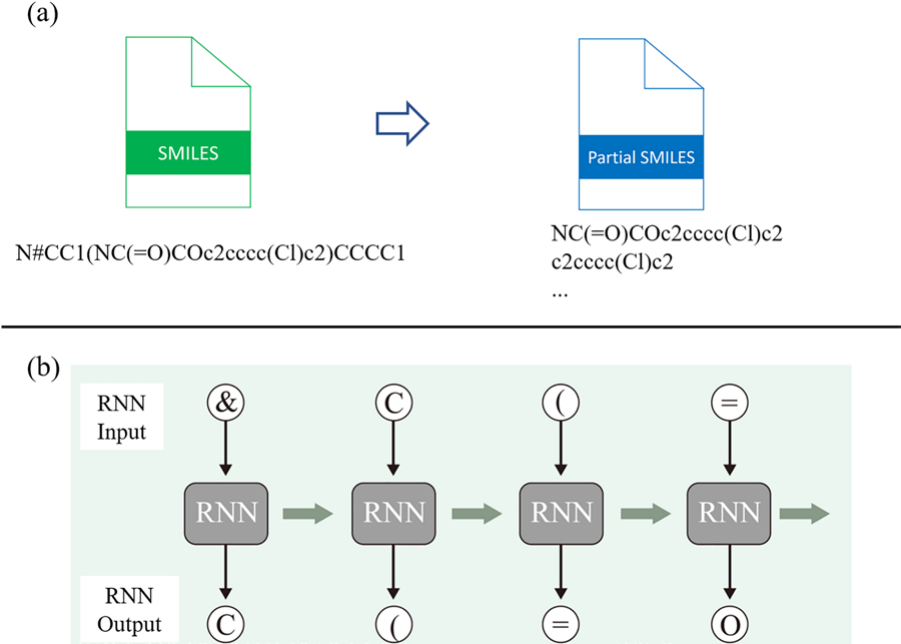
Details of RNN Model Training (**a**) Partial SMILES are extracted from complete SMILES. These partial SMILES datasets are used to train the RNN Model. **b** The RNN model predicts the next character from the previous string at each position

The role of RNN in MCTS is to select SMILES characters following an incomplete SMILES string in the expansion step and the default policy in the simulation step, as shown in Fig. 4(b). In the expansion step, the incomplete SMILES string $s_{1}s_{2}...s_{t}$ (encoded to $\varvec{x_0}\varvec{x_1}...\varvec{x_t}$ , e.g., one-hot vector) corresponding to path from root to the selected node is the input for RNN. RNN receives the encoded sequence as input and outputs the probability of selecting characters following the input sequence, which is incomplete SMILES. The probability of selecting a character $s^i$ based on output of RNN $\varvec{y_t}$ is as follows.

4 $$\begin{aligned} P(s^{i}_{t+1}|s_{1}s_{2}...s_{t}) = \frac{\exp (y^i_{t})}{\sum _j \exp (y^j_{t})} \end{aligned}$$

Several characters are selected through a fixed number of samplings from the probability and are added as child nodes to the selected node in the selection step. In the simulation step, a SMILES character that follows the current node is selected recursively until the terminal character is selected in the same way as the expansion step.

### RNN training

The role of MCTS is to generate partial SMILES string as described in above section. Therefore, RNN combined with MCTS should be trained with partial SMILES string rather than full SMILES. In this study, a dataset of partial SMILES strings was generated from a dataset of full SMILES, as shown in Fig. 4(a). Preprocessing was done as follows.

1. Partial SMILES strings were extracted exhaustively from full SMILES of the original dataset.
2. “Invalid” partial SMILES strings were filtered. Partial SMILES strings are regarded as valid if the SMILES generated by inserting partial SMILES strings into C*C or C(*)C is valid.

Approximately 250,000 molecules were obtained from the ZINC database to train the RNN model and evaluate the proposed generative model. 90 % of the molecules were used for training, and the remaining were used to evaluate molecule generation.

The RNN model consists of two layers of LSTM and receives encoded SMILES strings as input and outputs sequences of probability that represents the suitability of a SMILES character following the current input sequence for each position and each SMILES character in vocabulary. This model was trained on the preprocessed training set for 20 epochs using the Adam optimizer [34] to minimize cross entropy loss.

### Replacement of partial SMILES string

In the proposed method, the selection of partial SMILES strings removed from the initial point of SMILES is done in MCTS using the “Replacement” node. Fig. 3(b) shows how to select removed partial SMILES strings. Specifically, the “Replacement” node, which is a child node of the root node, has the values of the starting position of a removed SMILES string and its length of that. A partial SMILES string is generated from the grandchild nodes of the root node, and the generated string is replaced with the part of the initial point of SMILES corresponding to the “Replacement” node.

### Initial point of molecule

This approach is capable of generating new molecules from the original molecule. However, this approach has problem with generated molecules. Because this approach replaces the substructure generated by MCTS with a portion of the original molecule, the generated molecule is a molecule in which only one part of the original molecule has been changed on SMILES. In other words, the generated molecule is not modified in more than one place. Therefore, we propose a method that applies this approach multiple times (Fig. 3(c)). Specifically, for every fixed number of steps, the initial point of SMILES is replaced with the SMILES generated up to that point, and MCTS is performed from the beginning.

For efficient performance, it is important to know how the next initial point of SMILES is selected. In this study, we preferred to optimize SMILES with the maximum reward score, for example QED, LogP, etc. is selected as next initial point. The effect of the difference between fixed or changed initial points is investigated in the Experiment section.

This policy is simple and easy to understand and implement; however, it is possible that the generated molecules flow into a local solution. In addition, it is not necessary that future molecules that have desirable and better properties need to be generated from the best molecule among the generated molecules. Using this policy, good results are obtained in this study; however, the selection policy for next initial SMILES must be further considered to generate better molecules.

## Experiment

We conducted two experiments to demonstrate the performance of our method. The first experiment involves normal optimization, which modifies a molecule to maximize a single evaluation function. The optimization targets in this experiment were the QED [35] score and penalized LogP. QED is a measure of drug-likeness, and the more drug-like it is, the closer this value is to 1 ranging between 0 and 1. Penalized LogP is defined as follows.

5 $$\begin{aligned} PLogP(mol) =\, LogP(mol) + SAscore(mol) + RingPenalty(mol) \end{aligned}$$

The Penalized LogP consists of three terms: the normal LogP (octanol-water partition coefficient), the SA score that penalizes complex structures, and the penalties for large rings. Note that any other target property metrics that can be calculated from SMILES can also be used in this model. The 200 lowest PLogP/QED molecules in the validation data set ware selected as the seed in this experiment.

Molecule generation was done in 10,000 steps for each of the test molecules. We analyzed two cases for the proposed approach, depending on whether the seed molecule is fixed. The model in which the seed molecule is fixed is called “Single”, and the other model is called “Multi”. The seed molecule of the “Multi” model is replaced every 2000 steps with the highest scoring(PLogP/QED) molecule generated up to that point.

The second experiment is constrained optimization for comparison with conventional methods, which modifies a molecule to maximize a single evaluation function while satisfying some conditions. In this case, PLogP was optimized with the condition of using the Tanimoto coefficient based on ECFP4 fingerprint. Models perform 4 cycles $\times$50 steps =200 steps of optimization for each of the 800 molecules with the lowest PLogP in the ZINC dataset. Mol-CycleGAN [36] and GCPN [29] were used for comparison.

## Results

### Normal optimization

We evaluate the performance of the optimization task using the best molecular property score and the distribution of generated molecules using validity, uniqueness and novelty. Validity rate is defined as the ratio of SMILES that can be parsed by RDKit to all generated molecules. Uniqueness is the ratio of duplicate molecules to valid molecules, and Novelty is the ratio of molecules that are not included in the training dataset to those included.

**Fig. 5:**
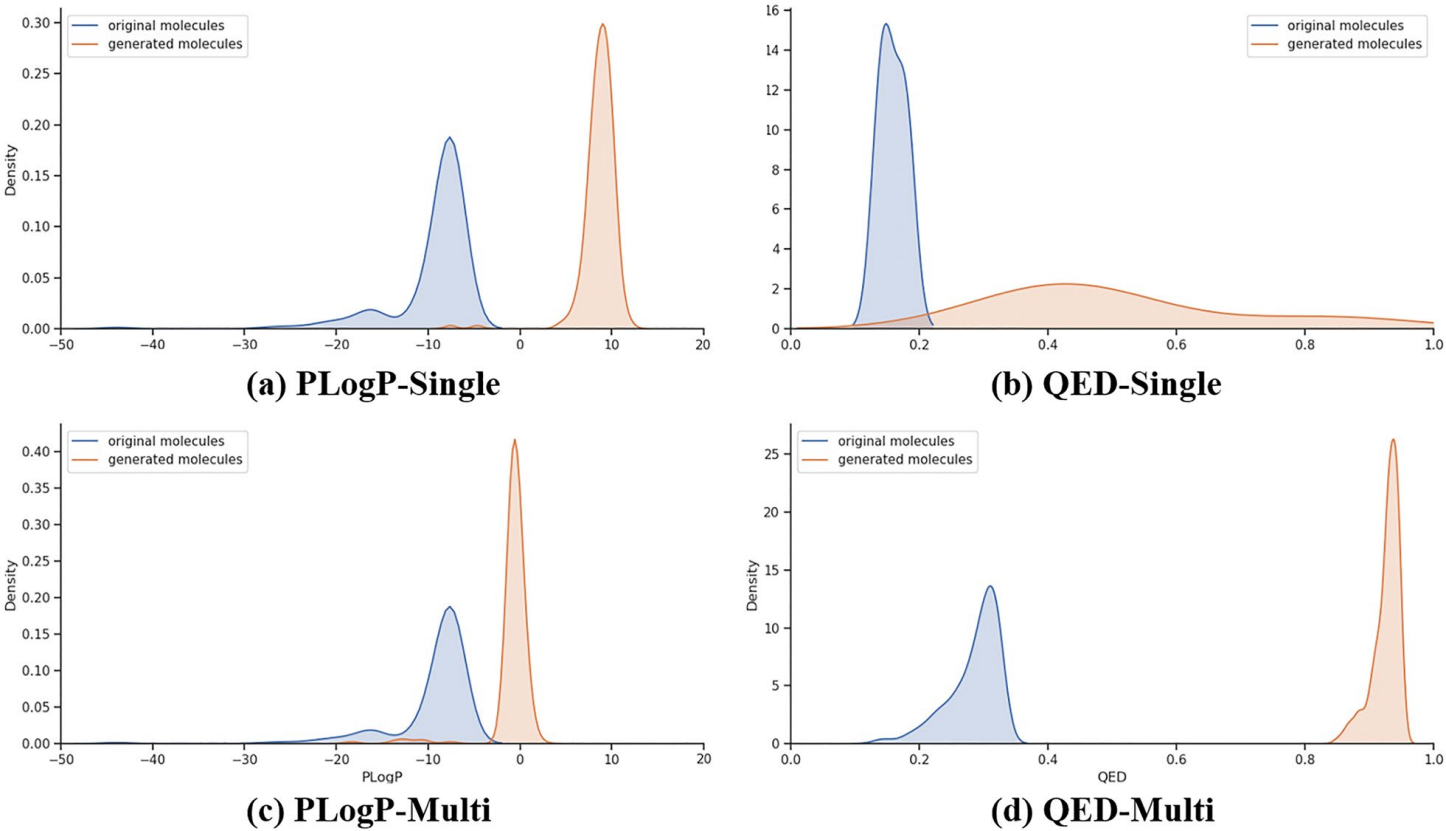
Distributions of generated molecules The distribution of the seed and generated molecules for each model and metrics. Blue: seed molecules, red: generated molecules

**Fig. 6:**
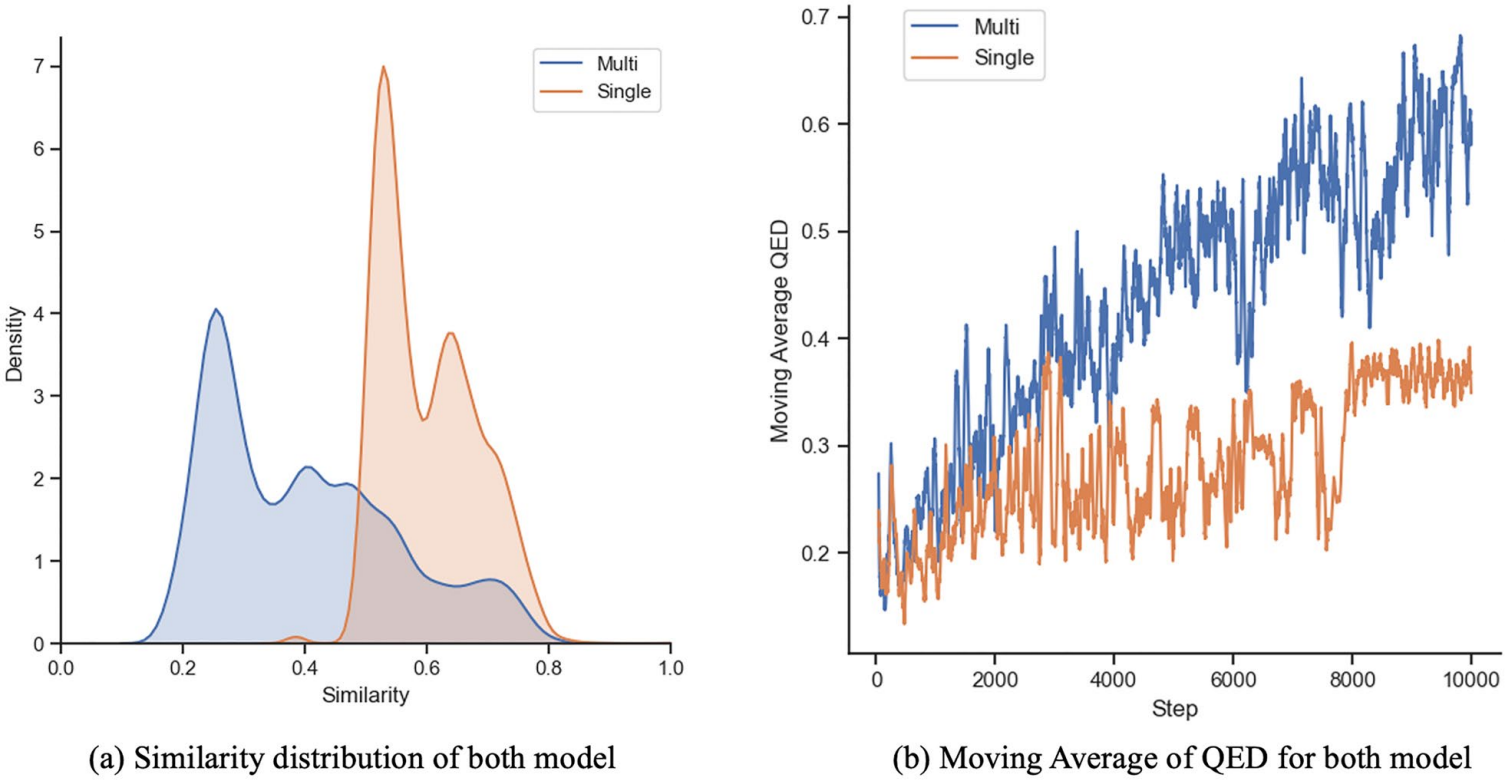
Example of optimization results for a molecule The optimization result from one randomly chosen compound. **a** The distribution of similarity to the seed molecule of generated molecules by our models. Blue: Multi model, red: Single model. **b** Moving average of QED with 20 steps. Blue: Multi model, red: Single model

**Fig. 7:**
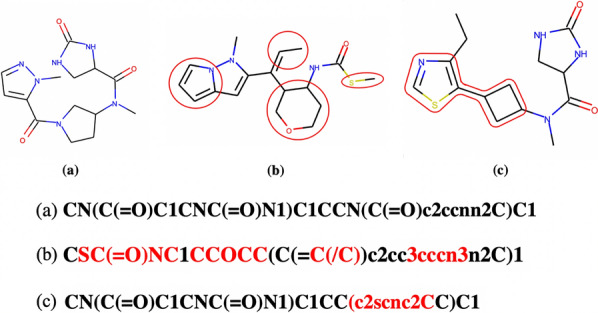
Differences in SMILES generated by each model Molecules generated by two models, showing the difference in terms of structure and SMILES. **a** Seed molecule. **b** Molecules generated by the “Multi” model and **c** molecules generated by the “Single” model. The red outlines in the molecular structures and the red character string in SMILES are changes from molecule (**a**). Note that deleted parts from the seed molecule during molecule generation are not highlighted in this figure

Optimization results are shown in Table 1. Our model shows sufficient results in both the QED and PLogP optimization tasks. In particular, the “Multi” model produces molecules with better scores than the “Single” model. This result is also shown in Fig. 5. The distribution of the QED/PLogP score of seed molecules shifted towards a higher score. This can be confirmed from Fig. 6, which shows that the “Multi” model generates molecules with higher scores as the number of steps increases. The distribution of similarity between the generated molecules and the seed molecules in Fig. 6 shows that the “Multi” model also generates molecules in regions where the “Single” model does not generate. Both the “Single” and “Multi” models generate molecules with relatively high similarity Additionally, the “Multi” model seeks higher-scoring molecules and expands the chemical space of generated molecules to a lower similarity region. The reason for this is that the “Multi” model can generate a molecule with changes occurring at multiple positions in SMILES as shown in Fig. 7 because the seed is updated, however, the “Single” model cannot generate such a molecule because it inserts partial SMILES at only one position.

**Table 1:** The results of the optimization tasks

Method	Penalized LogP				QED			
	1st	2nd	3rd	Validity	1st	2nd	3rd	Validity
ZINC	−9.41	−	−	−	0.285	−	−	−
Single	−2.20	−2.39	−2.53	29.8%	0.681	0.671	0.666	62.6%
Multi	11.33	11.20	11.10	31.8%	0.920	0.915	0.912	77.0 %

The mean of the top 3 highest scored Penalized LogP and QED scores of the validation set and the validity rate are described

**Fig. 8:**
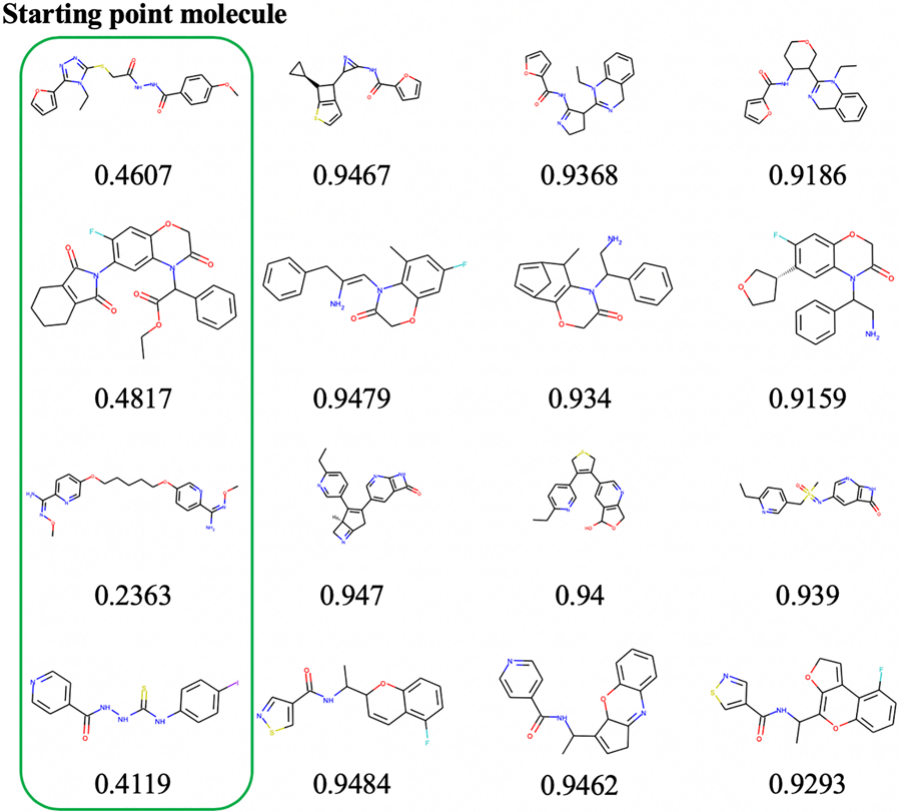
Structures of seed molecules and generated molecules In each row, molecule in the first column is the seed molecule, and generated molecules are shown in row. These molecules are generated by the “Multi” model that replaces seed molecules with generated molecules, with changing replace point at a certain number of steps. The QED score for each molecule is shown under the molecule

The results for the group of all generated molecules are shown in Table 2. Although validity is low, both uniqueness and novelty are high. The specific structures of the generated molecules are shown in Fig. 8. For each stating point molecule, molecules with high scores and different structures are generated. The values of the validity ratios differed considerably depending on the evaluation function. This is because the molecules generated by the PLogP optimization are larger; therefore, the search space is wider, and many invalid molecules are generated. In this manner, the evaluation function itself also affects the performance.

**Table 2:** Mean property of all generated molecules for 200 validation compounds

	Validity	Uniqueness	Novelty
PLogP-Single	0.298	0.981	1.0
PLogP-Multi	0.318	0.991	1.0
QED-Single	0.626	0.945	0.999
QED-Multi	0.770	0.958	0.999

Validity, uniqueness and novelty of all generated molecules are shown for four models. Validity is the ratio of valid SMILES to all generated SMILES. Uniqueness is the ratio of non-duplicate molecules to all valid molecules. Novelty is the ratio of molecules that are not included in the training dataset to all valid molecules

It is observed that the properties of molecules generated by the “Multi” model improve with each step. The number of steps for each iteration is 10,000, and the number of iterations is 5. We obtained good results from this experiment; however, these parameters are not optimum. Therefore, we need to investigate the relation between molecules and the hyperparameters of the “Multi” model, such as the number of steps and selection policy of the next seed molecule, in order to generate better molecules.

### Comparison with conventional methods of constrained optimization

The results of constrained optimization are shown in Table 3. The left column shows the difference in PLogP between the original molecule and the generated molecule with the highest PLogP as Improvement. Our method outperforms others in terms of the properties of molecules. However, success rate, which is the percentage of molecules with similarity above a threshold and improved PLogP, is worse than GCPN. Note that in GCPN, the policy is updated sequentially, while our method has a fixed policy. Thus, our method shows consistent results even when the number of evaluation steps is less (i.e., when optimizing properties that require considerable time to evaluate, such as DFT-based physicochemical values requiring several hours to calculate [37]).

**Table 3:** Results of constrained optimization for 800 validation molecules

$\delta$	GCPN			Mol-CycleGAN			MERMAID		
	Improvement	Similarity	Success (%)	Improvement	Similarity	Success (%)	Improvement	Similarity	Success (%)
0.2	4.12 ± 1.19	0.34 ± 0.11	100	5.79 ± 2.35	0.30 ± 0.11	93.8	9.94 ± 2.74	0.23 ± 0.04	100.0
0.4	2.49 ± 1.30	0.47 ± 0.08	100	2.89 ± 2.08	0.52 ± 0.10	58.8	6.04 ± 2.29	0.42 ± 0.02	100.0
0.6	0.79 ± 0.63	0.68 ± 0.08	100	1.22 ± 1.48	0.69 ± 0.07	19.3	1.99 ± 1.74	0.62 ± 0.02	85.3

The results of GCPN and Mol-CycleGAN are cited from Maziarka et al. [36]. The mean and standard deviation of improvement, similarity, and success rate of generated molecules are shown

## Discussion

In this experiment, we demonstrated that our method performs well in common tasks (QED and PLogP optimization with ZINC dataset and constrained similarity optimization); however, it can be used for various other tasks as well. To use this method, two areas require modification by the user: the evaluation function and the training data. For the evaluation function, all possible evaluation values that can be calculated from SMILES are available, and the user must design an appropriate evaluation function by combining these based on knowledge of the target task. The training data does not necessarily require modification by the user, as the purpose of training is to capture the features of partial SMILES and because the evaluation values are not directly related to training. Nevertheless, some types of tasks may bias the structure of relevant molecules; in such cases, optimization will improve efficiency if the user prepares the training data in advance.

As shown in the experimental results, our method produces molecules that improve the evaluation function value. Therefore, it is important to design the evaluation function carefully to avoid generating molecules that deviate from the seed molecule or have chemically unnatural structures.

Future work will focus on several approaches for fundamentally addressing these challenges, such as adding restrictions based on SMILES grammar to the substitution method. Molecular generation, starting from a specific molecule, has an important role in real-world applications; however, it has not been sufficiently studied compared with other types of molecular generation (generation from nothing). Hence, this study aimed to contribute toward filling this gap.

## Conclusions

In this paper, we developed a generative model based on MCTS and RNN to generate derivative molecules starting from a specific molecule. This model generates molecules by generating partial SMILES using MCTS and RNN and replacing it with part of SMILES of the seed molecule. In addition, we propose “Single” and “Multi” models. Unlike the “Single” model, the “Multi” model replaces the seed molecule with one of the generated molecules at a certain number of steps. As a result, it was demonstrated that the “Multi” model is superior to the “Single” model in terms of optimizing the QED score. Additionally, molecules generated by our model have high uniqueness and novelty, and the chemical space consisting of generated molecules is large in terms of similarity and molecular weight.

## Data Availability

MERMAID is freely available at https://github.com/sekijima-lab/mermaid.
